# Mechanism of Cordyceps sinensis and its Extracts in the Treatment of Diabetic Kidney Disease: A Review

**DOI:** 10.3389/fphar.2022.881835

**Published:** 2022-05-13

**Authors:** Wu Liu, Yiwei Gao, Yi Zhou, Fangning Yu, Xinyi Li, Ning Zhang

**Affiliations:** ^1^ Wangjing Hospital, China Academy of Chinese Medical Sciences, Beijing, China; ^2^ Department of Graduate Student, Beijing University of Chinese Medicine, Beijing, China

**Keywords:** *Cordyceps sinensis*, diabetic kidney disease, hyperglycemia, inflammation, oxidative stress

## Abstract

Diabetic kidney disease (DKD) is the major reason of chronic kidney disease (CKD)-caused end-stage renal failure (ESRF), and leads to high mortality worldwide. At present, the treatment of DKD is mainly focused on controlling the hyperglycemia, proteinuria, and hypertension, but is insufficient on the effective delay of DKD progression. *Cordyceps sinensis* is a kind of wild-used precious Chinese herb. Its extracts have effects of nephroprotection, hepatoprotection, neuroprotection, and protection against ischemia/reperfusion-induced injury, as well as anti-inflammatory and anti-oxidant activities. According to the theory of traditional Chinese medicine, *Cordyceps sinensis* can tonify the lung and the kidney. Several Chinese patent medicines produced from *Cordyceps sinensis* are often used to treat DKD and achieved considerable efficacy. This review summarized the clinical usage of *Cordyceps sinensis*, as well as its mainly biological activities including anti-hyperglycemic, anti-inflammatory, immunomodulatory, anti-oxidant, anti-fibrotic activities and regulation of apoptosis.

## Introduction

The prevalence of chronic kidney disease (CKD) is gradually increasing worldwide. If not treated timely, CKD may lead to heavy economic burden and poor prognosis by turning into end-stage renal failure (ESRF), which has no alternative treatment but renal replacement (Glassock et al., 2017). Diabetic kidney disease (DKD) has become the major reason of CKD-caused ESRF. DKD occurs in approximately 30% of patients with type 1 diabetes mellitus (T1DM) and 40% of patients with type 2 diabetes mellitus (T2DM), and about 50% of cases are in developed countries (Tuttle et al., 2014; Alicic et al., 2017). Moreover, DKD is often associated with cardiovascular disease and causes high mortality. Therefore, early diagnosis and treatment of DKD are highly essential. Several medicines are demonstrated to mitigate the progress of DKD *via* reducing the proteinuria, such as the renin-angiotensin system (RAS) antagonists, sodium glucose co-transporter 2 (SGLT-2) inhibitors, the glucagon like peptide-1 (GLP-1) receptor agonists (Mayer et al., 2019; Sarafidis et al., 2019; An et al., 2021; Wheeler et al., 2021). However, applications of these agents can be limited by their adverse effects. For example, RAS antagonists may raise the risk of hyperkalemia and hypotension, and SGLT2 inhibitors might result in mycotic genital infections, diabetic ketoacidosis, and acute kidney injury in patients who have volume depletion or borderline arterial pressure (Fitchett, 2019; Lu et al., 2019).

Increasing studies have been conducted on the pathogenesis of DKD over the past years, and it has been demonstrated that inflammation, oxidative stress, apoptosis, and fibrosis play vital role in the pathogenesis of DKD (Jha et al., 2016; Pichler et al., 2017; Xu et al., 2020). Applications of traditional Chinese medicine (TCM) in the treatment of DKD have become hot topics because of its advantage of muti-target therapy, which can reduce the side effects caused by other agents and increases efficacy. Based on it, TCM is used as an adjunctive treatment for DKD, improving the symptoms in patients with DKD, and alleviating the inflammation, oxidative stress, renal fibrosis, and so on (Ekor, 2014; Lu et al., 2019).

**FIGURE 1:**
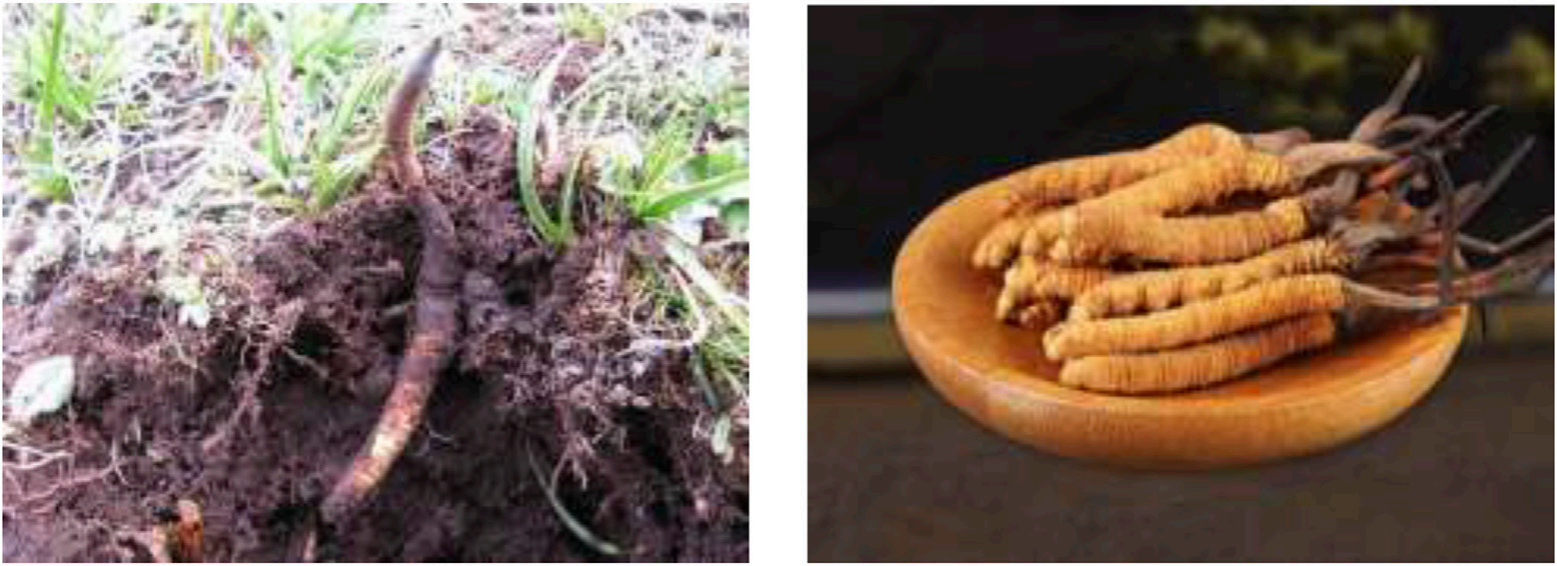
*Cordycepin sinensis* in the soil (left). Traditional Chinese herb *Dong Chong Xia Cao* decoction pieces (right).

*Cordyceps sinensis (BerK.) Sacc* (Figure 1), also called *Cordyceps sinensis* (*C. sinensis*), is a kind of ascomycetes parasitic fungi which belongs to family *clavicipitaceae*, and it is mainly parasitic on insects and other arthropods (Olatunji et al., 2018). In China, *C. sinensis* is a precious Chinese herb with a medical history of hundreds of years. *C. sinensis* is first mentioned from *Ben Cao Bei Yao* (written by *Wang Ang,* tracing back to 1694AD). *C. sinensis* is wildly used in clinic, especially for nourishing the kidney and tonifying the lung. Furthermore, several medicinal preparations produced from *C. sinensis* are extensively used as auxiliary agents for the clinical treatment of DKD (Kai et al., 2015; Sheng et al., 2020). Nowadays, increasing researches have identified the bioactive ingredients in *C. sinensis* which can induce various effects on the treatment of DKD, such as anti-hyperglycemia, anti-inflammatory, anti-oxidant activities (Kiho et al., 1993; Shin et al., 2009b; El Zahraa Z El Ashry et al., 2012; Xiao et al., 2012). There are many mechanisms supporting the role of *C. sinensis* for the treatment of DKD. Therefore, keeping in view of the above facts, this review summarizes the recent studies pertaining to the chemical constituents, pharmacology, and toxicity of *C. sinensis*, and the biological activities of *C. sinensis* in DKD treatment to provide evidence for better clinical use of *C. sinensis*.

## 2 Toxicity, Chemical Constituents, and Pharmacology

The genus of *cordyceps* is abundant. More than 400 species of *cordyceps* distributed widely, mainly in North America, Europe, East and Southeast Asia. At present, some studies have demonstrated that *C. sinensis* has a variety of pharmacological effects, such as nephroprotective, hepatoprotective, neuroprotective, anti-inflammatory, and anti-oxidant activities (Yue et al., 2013; Cao et al., 2020). Due to the harsh environment in which it grows, *C. sinensis* is rare and precious, so it is important to explore its main active ingredients for better utilization.

### 2.1 The Safety and Toxicity of *Cordyceps sinensis*

*C. sinensis* is widely used as the medicinal plant and health food. For the moment, there is still debate about whether we should worry about the toxicity of *C. sinensis.* The most prominent concern is about the accumulation of heavy metals, especially arsenic (As) in *C. sinensis* due to the impact of global environmental pollution. In 2016, it was warned by the National Medical Products Administration (NMPA) that long-term use of *C. sinensis* may lead to health risks, based on that total As in *C. sinensis* and its related products is 4.4–9.9 mg/kg (Li Y. et al., 2019a). However, As in *C. sinensis* is hard to be absorbed by human body (Liu et al., 2016). Besides, limited intake of *C. sinensis* cannot cause significant harm to human body (Zhou et al., 2018). Generally, the recommended dose of *C. sinensis* should not exceed 4 g per day and should not be used for more than 5 months per year (Li Y. et al., 2019a). Based on the existing studies, a clinical use of *C. sinensis* 3–6 g/day in patients with chronic renal failure was safe and could slow the progression of renal function (Zhu et al., 1998). Allen et al. found that daily taken 3.15 g of the synthetic compound of *C. sinensis* for 5 weeks had no additional adverse reactions compared with placebo (Parcell et al., 2004). Moreover, cultured *C. sinensis* has higher controllability on their growth environment, so it has certain advantages over natural *C. sinensis* in the control of heavy metal content, and Li et al. used the xanthine oxidase assay, the induction of hemolysis assay and the lipid peroxidation assay to confirm the strong anti-oxidant activity of both natural *C. sinensis* and cultured *Cordyceps* mycelia (Li et al., 2001), suggesting that natural *C. sinensis* can be replaced by cultured *C. sinensis* (Liu et al., 2016). So, *C. sinensis* is considered to be safe. In brief, the toxicity of *C. sinensis* is controllable by rational intake and replacement by cultured ones.

### The Main Chemical Constituents and Pharmacology of *Cordyceps sinensis*

A variety of *C. sinensis* derivatives have been applied to the clinic. The strains isolated from *C. sinensis* have been artificially cultivated and fermented into various products are in clinical use in China (Yang et al., 2018). There are 9 kinds of productions, among them, Jinshuibao capsule, Bailing capsule, and Zhiling capsule are wildly used to ameliorate the kidney function in patients with chronic renal failure, renal transplant and DKD (Wang et al., 2013; Zhang et al., 2017; Lu et al., 2018; Yang et al., 2018). Many active components have been identified from *C. sinensis*, including cordycepin, adenosine, sterols, and many polysaccharides (Liu et al., 2015; Olatunji et al., 2018; Ashraf et al., 2020). The main biological effects of those active components are concluded in Table1. Cordycepin (3′-deoxyadenosine) is considered as the most representative bioactive constituents and the marker of the quality of *C. sinensis* (Shashidhar et al., 2013). Cordycepin is a nucleoside analogue composed of a purine molecule linked to a ribose sugar, with a content ranged from 0.0076% to 0.029% (w/w) in *C. sinensis* (Hu et al., 2015). Cordycepin is involved in the process of transcription and activation of polymerases, exerting the anti-hyperglycemia, anti-inflammatory, and immunomodulatory effects *via* interfering in mTOR signal pathway, then repressing apoptosis and alleviating the kidney injury in DKD (Tuli et al., 2014; Yoon et al., 2018; Ashraf et al., 2020).

**TABLE 1:** The main active extracts in Cordyceps Sinensis.

Classification	Constituent	Therapeutic Effect	Reference
Nucleosides	Cordycepin	Ant-itumor, anti-inflammatory, anti-microbial, anti-hyperglycemia, inhibition of platelet aggregation, hypolipidemic, analgesic, immunomodulatory, antileukemic, neuroprotective activities	Ashraf et al. (2020)
	Adenosine	Anti-convulsant, anti-inflammatory, inhibition of neurotransmitter release, modulation of adenylate cyclase, immunomodulatory, cardioprotection activities	Shashidhar et al. (2013)
	Guanosine	Immunomodulatory effect	Kim, (2010)
	Cordysinin A-E	Cerebroprotective, superoxide anion inhibition, anti-inflammatory effects	Chen et al. (2013)
Polysaccharides	Exopolysaccharide fraction (EPSF)	Anti-tumor, anti-oxidant, anti-inflammatory, Immunomodulatory activities	Zhang et al. (2005a)
	Acid polysaccharide (APS)	Anti-oxidant, Immunomodulatory effects	Chen et al. (2010)
	CPS-1	Anti-oxidant, anti-inflammatory, immunomodulatory, metabolic regulation, nephroprotective, anti-hyperglycemia activities	Kwon et al. (2001)
	CPS-2	Cell proliferation inhibition, protection of chronic renal failure	Wang et al. (2014)
	Mannoglucan	Anti-tumor, cytotoxicity activity	Wu et al. (2007)
	CME-1	Anti-oxidant activity, platelet activation	Chang et al. (2015)
	PS-A	Inhibitory activity against cholesterol esterase	Kim, (2010)
	Cordyglucan	Anti-tumor, immunomodulatory activities	Yalin et al. (2005)
	D-mannitol (Cordycepic acid)	Anti-inflammatory, anti-fibrosis, diuretic, improve the plasma osmotic pressure, anti-free radical, anti-tussive activities	Hu et al. (2015)
Sterols	Ergosterol	Anti-tumor, cytotoxicity, antimicrobial activities	Matsuda et al. (2009)
	H1-A	Inhibit mesangial proliferation, regulation of apoptosis, immunomodulatory, reduce the production of anti-dsDNA	Yang et al. (2003)
	β-sitosterol	Anti-tumor, anti-hyperglycemia, hypolipidemic, anti-arthritic, hepatoprotective activities	Ponnulakshmi et al. (2019)
	Cerevisterol	Anti-inflammatory, anti-oxidant, anti-microbial, anti-hypoxia activities	Wang et al. (2019)
	5α,8α-epidioxy-24(R)-methylcholesta-6,22-dien-3β-D-glucopyranoside and 5α,6α-epoxy-24(R)-methylcholesta-7,22-dien-3β-ol	Anti-tumor	Chen et al. (2013)
Amino acids and Polypeptides	Cordymin	Anti-inflammatory, anti-nociceptive, using for diabetic osteopenia	Qian et al. (2012)
	Cyclodipeptides A	Anti-malarial, cytotoxic activities	Jia et al. (2005)
	Cordycemides A and B	cytotoxic activity	Jia et al. (2009)

Adenosine is another main bioactive constituents of *C. sinensis*. The best-known effects of adenosine are anti-inflammatory and anti-convulsant activities (Shashidhar et al., 2013). Adenosine exerts biological effects and signal transduction *via* binding to adenosine receptors distributed on cell membranes, which are widely expressed in metabolic regulatory organs and kidney (Oyarzún et al., 2017). Adenosine plays a critical role in regulating glomerular perfusion pressure, filtration rate and renal tubular reabsorption, as well as influencing insulin secretion and regulating glucose homeostasis (Peleli and Carlstrom, 2017). Modulating adenosine receptor signal transduction is a new strategy for the treatment of DKD. Adenosine contributes to protecting DKD-related renal insufficiency by reducing the production of pro-inflammatory cytokines (e.g. TNF-α, IL-12, INF-γ) and increasing the expression of anti-inflammatory cytokines, such as IL-10 (Haskó et al., 1996). Although there is still ongoing research about its anti-oxidant activities. Previous study have shown that adenosine could prevent oxidative stress damage to cells by inducing an increase of glutathione peroxidase 1 (Zhang Y. et al., 2005b).

Polysaccharides are important and complex active components of *C. sinensis*, and the different types of polysaccharides separated from *C. sinensis* also have the activities of anti-inflammatory, anti-oxidant, metabolic regulation and so on (Paterson, 2008; Yan et al., 2014). CPS-1, a polysaccharide constituent detected in *C. sinensis*, is reported to has an insulin-like effect and promote the secretion of insulin, and it is expected to be developed as an agent to anti-diabetes (Kwon et al., 2001). Besides, various polysaccharides (such as EPS, CPS-F) exert anti-inflammatory and anti-oxidant activities *via* inhibiting the expression of pro-inflammatory cytokines, promoting the secretion of anti-inflammatory cytokines and scavenging free radicals to slow the progression of DKD (Wang et al., 2015; Li et al., 2020). In addition to focusing on a single component, it is also deemed that the effect of *C. sinensis* comes from a combination of various components. The anti-oxidant activity of *C. sinensis* may be related to the combined effects of polysaccharides, flavonoids, and polyphenols (also called the hydrogen-donating anti-oxidants) (Li et al., 2001; Yu et al., 2006). In order to clarify the mechanism of *C. sinensis*, further exploration is needed in the future.

## 3 The Biological Activities of *Cordyceps sinensis* in Treating Diabetic Kidney Disease

Persistent hyperglycemic environment enhances the protein glycation reaction, and increase of advanced glycation end products (AGEs), which can stimulate the production of reactive oxygen species (ROS) (Abou-Hany et al., 2018). Accumulated ROS induces oxidative stress and inflammation, meanwhile activates various signaling pathway, such as mitogen-actived protein kinases (MAPKs) and nuclear factor-κB (NF-κB) pathway, which in turn aggravated oxidative stress and inflammation (Tomás et al., 2002; Abou-Hany et al., 2018, Jia et al., 2018, Yan et al., 2020). Besides, excessive ROS could attack the unsaturated fatty acids in the biofilm phospholipid bilayer, causing lipid peroxidation and insulin resistance and then triggering multiple cascading reactions (Wei et al., 2019). The role of the signal mechanism in the treatment of DKD by *C. sinensis* will be discussed separately below.

### 3.1 Anti-Hyperglycemic Activity

The most important target for DKD treatment is to control hyperglycemia as early as possible. Long-term hyperglycemia is considered to be the main reason for abnormalities of tissue and microvascular. The role of *C. sinensis* in the treatment of DKD has been demonstrated (Luo et al., 2015). Jinshuibao capsule, a kind of *cordyceps* preparation, had additional beneficial effects on the DKD patients treated with angiotensin receptor blockers (ARB), and ameliorating the outcomes of patients (Cao et al., 2007). Several active constituents contribute to the anti-hyperglycemic activity. High fiber in the fruiting body of *C. sinensis* could improve glucose intake and insulin resistance of cells, which might due to its influence on glucose absorption (Lo et al., 2004). Moreover, polysaccharides are also the pivotal active constituents in anti-hyperglycemia. Polysaccharides-enriched extract could lower glycemic levels by 60–70% (Zhang et al., 2006). CS-F10, a polysaccharide derived from *C. sinensis* hot water extracts, significantly reduced the glucose levels both in normal and hyperglycemic mice by increasing the activity of glucokinase in livers (Kiho et al., 1999).

Besides, metabolic factors such as insulin levels and resistances may also be the central factors in DKD (Reidy et al., 2014). Insulin resistance can act on the glomeruli by mediating metabolism and hemodynamics, resulting in intraglomerular hypertension and thus influencing the progression of DKD (Karalliedde and Gnudi, 2016). Moreover, insulin resistance can also lead to the high salt-sensitivity, which associates with albuminuria and impairment of renal function, increasing the risk of macrovascular dysfunction (Karalliedde and Gnudi, 2016). Therefore, boosting insulin sensibility is imperative. It was shown that both the fermentation mycelia and the fermentation liquid of *C. sinensis* could significantly improve glucose response in OGTT and boost the insulin level in nicotinamide and streptozotocin (STZ)-induced diabetic rats (Lo et al., 2006). Several studies demonstrated that polysaccharides in *C. sinensis* could significantly decrease the blood glucose, and increase the insulin sensitivity in diabetic models (Kiho et al., 1993; Lo et al., 2006). In the state of insulin resistance, the impairment of insulin signaling in mesangial cells (MCs) might cause hypertrophy, proliferation, and matrix deposition of MCs (Artunc et al., 2016). Normally, relying on the actin cytoskeleton of podocytes, the glucose transporter 4 (GLUT4)-rich vesicles can be translocated, which helps the insulin-induced glucose uptake, while the destruction of insulin signaling leads to damage of the actin cytoskeleton, and the GLUT4 nuclear translocalization function is missing, eventually contributing to the loss of podocytes and the production of proteinuria (Coward et al., 2005; Artunc et al., 2016). β-sitosterol, an effective anti-oxidant and anti-hyperlipidemia plant sterol in *C. sinensis*, was reported to lower lipid levels, restore insulin sensitivity, and activate insulin receptors and GLUT4 in adipose tissue and eventually exert anti-hyperglycemia activity in diabetic rats (Ponnulakshmi et al., 2019). Cordycepin also plays a vital role in attenuating insulin resistance. It could improve insulin sensitivity by monitoring serum insulin levels and homeostasis model assessment of the insulin resistance index (Niu et al., 2010).

In addition, pathogenic oxygen free radicals caused by hyperglycemia play an important role in the pathogenesis of DKD (Sailaja Devi et al., 2000). CSP-1, a polysaccharide which was separated from *C. sinensis*, had the effects of scavenging free radicals, and exerted anti-hyperglycemia effect *via* significantly decreasing the level of glucose by inducing the release of insulin in the remainder pancreatic cells and/or reducing the insulin metabolism (Li et al., 2006). The metabolic process of glucose involves varieties of reactions and participations of cytokines. Among them, as an important endocrine organ, adipose tissue is closely related to inflammation and insulin resistance by secreting various hormones, pro- and anti-inflammatory adipokines, which can affect glucose, lipid metabolism and energy homeostasis (Snel et al., 2012). Patients with T2DM have more visceral subcutaneous fat than normal individuals, hyperglycemia causes macrophages in adipose tissue to secrete pro-inflammatory factors, and stimulates the accumulation of ectopic fat, so that the secretion of pro- and anti-inflammatory adipocytokines is imbalanced (Snel et al., 2012). The increase of pro-inflammatory adipocytokines, such as leptin, can aggravate inflammation *via* stimulating the secretion of inflammatory factors (TNF-α, IL-6, IL-12), and the increase of resistin reduces the anti-inflammatory effect of adiponectin, meantime inducing the aggravation of insulin resistance (Tilg and Moschen, 2006). Using *C. sinensis* could significantly improve the insulin sensitivity and boost the expression of adiponectin within plasma and adipose tissue in STZ-induced diabetic mice (Huang et al., 2016). Thus, *C. sinensis* might become an effective adjuvant medicine in the treatment of DKD by its anti-hyperglycemic function and improving insulin resistance.

### 3.2 Anti-Inflammatory Activity

Chronic low-grade inflammation, also known as metaflammation, is a factor associated with the initiation and development of DKD. Metaflammation activates macrophages, and then secretes cytokines such as TNF-α, IL-1β to regulate immunity, afterwards the excessive activation of immune cells will also stimulate the inflammatory response (Li et al., 2020). There is a causal relationship between inflammation and insulin resistance, pro-inflammatory cytokines have been shown to mediate insulin resistance (Hotamisligil et al., 1993; Rotter et al., 2003). A meta-analysis suggested that *C. sinensis* combined with ACEI/ARB in patients with III to IV stage of DKD was effective to alleviate proteinuria, inflammation, and dyslipidemia by comparing 24-h proteinuria, urinary albumin excretion rate, microalbuminuria, serum creatinine, blood urea nitrogen, C-reactive protein, serum triglycerides, total cholesterol (Luo et al., 2015). Recent DKD studies have focused more on inflammation, thus studying the anti-inflammatory mechanisms of TCM has also provided a new perspective on treatment progress. The extracellular polysaccharides (EPS) extracted from *C. sinensis* mycelial fermentation could suppress the inflammatory NF-κB pathway by inhibiting the expression of pro-inflammatory cytokines TNF-α and iNOS, and improving the expression of the anti-inflammatory regulator IL-10 in LPS-induced mice models (Li et al., 2020).

NADPH oxidase (NOX) plays a prominent role in inflammation, and is one of the main sources of ROS in biological systems (Gorin and Wauquier, 2015). As one of the members of NOX family, NOX4 is highly expressed in kidney, and recently, NOX1 has also been found in kidney (Sedeek et al., 2013). Exposure to hyperglycemia, the expression of NOX4 in mouse proximal renal tubular cells is increased, using NOX1/4 inhibitors (GKT136901) or NOX4 siRNA could block the damage of hyperglycemia to kidney, and reduce the production of proteinuria (Sedeek et al., 2013). Cordycepin could reverse the albumin-induced epithelial-to-mesenchymal transition (EMT) in HK-2 cells *via* reducing the expression of Rac1, NOX4, p22phox, p47phox, and inhibiting the formation of ROS to alleviate inflammation and oxidative stress (Xiao et al., 2012). TNF and TNF receptor-1 (TNFR1) participate in the recruitment of NOX1, which is involved in inflammation, cell proliferation, and immune defense, the binding of TNF homotrimer to TNF-R1 initiates the binding of the adaptor protein, then recruits other effector proteins to form the TNF-R1 signaling complex, leading to the activation of multiple pathways, and then producing ROS to initiate inflammation (Chen and Goeddel, 2002; Kim et al., 2007). Based on a network pharmacological study, TNF is the crucial target in the treatment of DKD by *C. sinensis* (Li et al., 2021). And CPS-F, which was extracted from *C. sinensis*, could reduce the expression of pro-inflammatory cytokines and the production of ROS in human MCs by relying on TNF-R1, and inhibiting the activity of NOX1 to exert anti-inflammatory activity (Wang et al., 2015).

In recent years, it has been demonstrated that the activation of purinergic 2X7 receptor (P2X7R) and nucleotide-binding oligomerization domain-like receptor protein 3 (NLRP3) inflammasome in podocytes are associated with the pathogenesis of DKD (Vonend et al., 2004; Shahzad et al., 2015). Robert et al. identified that P2X7R was highly expressed in renal biopsy specimens from patients with DKD, and the expression rate of glomerular P2X7R was about 50% (Menzies et al., 2017). The activation of P2X7R on M1 polarized pro-inflammatory macrophages, inducing the assembly of NLRP3 inflammasome, and then resulting in the release of pro-inflammatory factors and generation of inflammation (Olefsky and Glass, 2010). Therefore, antagonizing P2X7R may become a new type of anti-inflammatory therapy. Extensive evidence on *C. sinensis* involved improving DKD *via* inhibiting P2X7R/NLRP3 inflammasome axis activation. It could down-regulate the expression of P2X7R, and NLRP3 inflammasome (including NLRP3, ASC, caspase-1) *in vivo* and *in vitro* experiments, and the expression of downstream factors IL-1β and IL-18 was also significantly decreased, *C. sinensis* exerted an anti-inflammatory effect by antagonizing the P2X7R/NLRP3 inflammasome axis to relieve podocytes damage in DKD (Wang et al., 2018). *C. sinensis* can alleviate inflammation in varieties of ways to delay the progression of DKD, and the active constituents contained in it are vital to the anti-inflammatory agents in the pipeline.

### 3.3 Immunomodulatory Activity on Macrophages

*C. sinensis* regulates immunomodulatory activity to improve DKD may be primarily concentrated on its polysaccharides and nucleoside components. The immunomodulatory effect of natural plant polysaccharides has been recognized (Yan et al., 2014). The activation of macrophages is an important event in immune response, and the infiltration of macrophages is related to the progression of DKD to ESRF (Tang and Yiu, 2020). Plant polysaccharides can regulate the balance of pro- and anti-inflammatory cytokines secreted by macrophages to normalize the immunity (Műzes et al., 2012; Yin et al., 2019). Polysaccharides activate macrophages by binding to specific receptors on the surface of macrophages, then initiating a series of intracellular signaling cascade reactions. They exert immunomodulatory effects by regulating the production of ROS, secretion of cytokines and chemokines, cell proliferation and phagocytic activity of macrophages (Yin et al., 2019). Activated macrophages can be polarized into classically activated M1 and alternatingly activated M2 forms, where M2 cells have anti-inflammatory activity, abrogate the Th1 response, promote the Th2 response, release inflammatory substances, and promote epithelial and vascular repair (Belska et al., 2010; Wada and Makino, 2013). *C. sinensis* could promote macrophages polarize towards an M2 phenotype to inhibit the inflammatory response, and regulate the production of cytokines in RAW264.7 cells through the MAPK and PI3K/Akt signaling pathways, exerting immunomodulatory activity on macrophages (Liu et al., 2021). The balance of pro- and anti-inflammatory activities exerted by M1/M2 macrophages plays a pivotal role in the regulation of immune system (Mantovani et al., 2007), cordycepin and adenosine could alter the phenotypic conversion of macrophages by reducing the expression of pro-inflammatory factors and chemokines, such as IL-1β, IL-6, TNF-α, RANTES, CX3CL1, and increasing the expression of anti-inflammatory cytokines, such as IL-1ra, IL-10, TGF-β (Shin et al., 2009b). By the way, the polarization of M2 macrophages can induce the occurrence of EMT and fibrosis, and M2 macrophages can promote the secretion of TGF-β, which is generally recognized as the typical pro-fibrosis factor and an anti-inflammatory factor (Zhu et al., 2017; Srivastava et al., 2019). *C. sinensis* up-regulated the mRNA levels of anti-inflammatory mediator (IL-10), and suppressed the mRNA levels of pro-inflammatory mediator (IL-1β) and the production of NO in LPS-induced RAW264.7 cells, regulating pro- and anti-inflammatory mediators to balance M1/M2 macrophages and avoiding excessive polarization of macrophages to exert immunomodulatory effect (Liu et al., 2021). It was demonstrated that cordycepin could inhibit the expression of TNF-α, IL-6, IL-1β, and mediate the immune responses in macrophages, decrease the expression of diabetes regulating genes (11β-HSD1, PPARγ) in activated macrophages, finally play the immunomodulatory effect (Shin et al., 2009a). In summary, *C. sinensis* is closely linked to anti-inflammatory activity in regulating the immunity of macrophages. The *C. sinensis* preparations and their extracts are expected to become potential therapeutic agents for regulating metabolic inflammatory diseases.

Under the guidance of TCM theory, as a tonic, *C. sinensis* may also have a certain relationship with its regulation of T cells. The balance between effector T cells and regulatory T cells plays a regulatory role in the pathogenesis of DKD (Sabapathy et al., 2019). Lo et al. found that the polysaccharides extracted from the fermented mycelium of *C. sinensis* could increase the weight of the thymus gland in diabetic rats, and enhance the immunity (Lo et al., 2006). One study has reported that *C. sinensis* could regulate the ratio of CD4^+^CD25^+^FoxP3^+^ regulatory T (T reg) cells and Th17 cells in spleen and pancreatic lymph nodes of non-obese diabetic mice, thereby inhibiting DM (Shi et al., 2009). However, the regulation effect of *C. sinensis* on the balance of T cells in DKD is less, and further researches remain to be studied.

### 3.4 Anti-Oxidant Activity

Due to long-term exposure to high glucose environment, excessive amounts of superoxide, NO and other reactive oxygen substances are produced in cells, resulting in the decrease of endogenous and exogenous anti-oxidants, which cannot interact with ROS to counteract oxidative damage of cells (Di Vincenzo et al., 2019; Urner et al., 2020). The overproduction of ROS contributes to the microvascular damage, combines with oxidative stress, inducing the cellular apoptosis in DKD (Di Vincenzo et al., 2019). Hence, it is crucial to reduce the production of ROS by using anti-oxidant agents. It has been reported that *C. sinensis* has anti-oxidant effect (Dong and Yao, 2008). Fatma et al. found that *C. sinensis* could exert powerful anti-oxidant effect to significantly reduce the pancreatic malondialdehyde levels and increase the serum anti-oxidant property *in vitro* and *in vivo* experiments (El Zahraa Z El Ashry et al., 2012). And the anti-oxidant effect of *C. sinensis* might due to inhibit the production of ROS *via* the interference of *C. sinensis* extracts in ERK and Akt pathways (Wang et al., 2015).

ROS can induce the influence of unsaturated fatty acids, and then induce the occurrence of lipid peroxidation, subsequently resulting in a decrease in fluidity of podocytes membranes, eventually with consequent damage of the structures and functions of the cell membranes (Li et al., 2001; Su et al., 2019; Sidhom et al., 2021). The extracts of *C. sinensis* could attenuate lipid peroxidation and scavenge free radicals, while the enriched fraction of polysaccharides had even stronger anti-oxidant activity (Li et al., 2001). A current study indicated that the anti-oxidant activity of *C. sinensis* was achieved by melanin, it could scavenge DPPH• and the chelating ability on ferrous ions (Dong and Yao, 2012). A study found that the phenols and flavonoids contained in Indian Himalayan isolate of *C. sinensis* (AECS) maybe the main components to exert anti-oxidant activity, AECS could significantly inhibit the release of TNF-α and IL-1β, and suppress the production of NO in LPS-induced THP1 cells (Rathor et al., 2014). Moreover, the rich unsaturated fatty acids in low-density lipoprotein (LDL) are susceptible to oxidative degeneration under cyclic oxidative conditions, and *C. sinensis* could reduce the occurrence of cardiovascular events in DKD by protecting the oxidation of LDL (Yu et al., 2006).

There were accumulating evidence demonstrated that excessive albumin could activate NOX *via* Rac1, thus stimulating the production of ROS, and overproduction of ROS and NOX maybe take part in EMT induced by albumin (Whaley-Connell et al., 2007; Xiao et al., 2012). Cordycepin could suppress oxidative stress damage and protect HK-2 cells by significantly inhibiting the expression of NADPH system constituent proteins, the production of ROS and the activity of NOX (Xiao et al., 2012). *C. sinensis* can scavenge free radicals and relieves the hyperactive oxidative systems in oxidative stress to protect the impaired kidney.

### Anti-Fibrotic Activity

Renal interstitial fibrosis (RIF) is the ultimate outcome of DKD. The interaction of hemodynamic and metabolic factors leads to increased intraglomerular pressure and actives vasoactive hormone pathway, which activates the secretion of proliferation and fibrosis-promoting cytokines, such as prosclerotic cytokine, TGF-β and the permeability enhancing growth factor, vascular endothelial growth factor (VEGF), ultimately leads to changes in kidney permeability and accumulation of extracellular matrix (ECM) and results in fibrosis (Zatz et al., 1986; Soldatos and Cooper, 2008). EMT plays an imperative role in the progress of fibrosis formation, and TGF-β is the key promoter of EMT. TGF-β induces the tubular epithelial cells turn into myofibroblasts, chronic inflammation, and leads to the production and accumulation of ECM (Gu et al., 2013; Kanasaki et al., 2013). At present, the anti-fibrotic treatment of *C. sinensis* mainly focused on regulating EMT and TGF-β. The nucleoside/nucleobase-rich extract from *C. sinensis* could significantly alleviate the development of DKD by inhibiting the progression of EMT and the accumulation of ECM *via* suppressing p38/ERK signaling pathway, together with decreased expression of fibrosis-related protein such as fibronectin and collagen Ⅰ (Dong et al., 2019). Similarly, *C. sinensis* could mitigate the fibrosis by inhibiting the expression of Bcl-2-associated athanogene 3 (BAG3) and α-SMA in unilateral ureteral obstruction (UUO) rats, and regulating the formation of EMT (Du et al., 2015).

As an anti-inflammatory factor, TGF-β1 is also a typical pro-fibrotic factor and involved in the TGF-β/Smad signaling pathway, regulating the transcription of TGF-β1-reactive genes, interruption of any step in this cascading process may lead to the blocking of TGF-β1 signaling pathway, which leads to the blocking of myofibroblast activation (Wotton and Massagué, 2001). Hence, the agents for blockage TGF-β1/Smad signaling pathway are important to alleviate fibrosis. *C. sinensis* could exert anti-fibrotic activity by mediating TGF-β1/Smad signaling pathway, and attenuate renal fibrosis in 5/6 nephrectomy rats by abrogating the expression of TGF-β1, Smad2/3, TβRⅠ, TβRⅡ, p-Smad2/3, α-SMA, and FSP1, and upregulated Smad7 to inhibit EMT (Pan et al., 2013). As mentioned above, 3′-deoxyadenosine, also known as cordycepin, was identified as the mainly anti-fibrotic active biology constituent in *C. sinensis*, acting by interfering with profibrotic Smad signaling *in vitro* and *in vivo* (Gu et al., 2013). Another study indicated that cordycepin could effectively upregulate the anti-fibrosis factor hepatocyte growth factor (HGF) at both the gene and protein levels in NRK-49F cells to mediate the blockage of Smad2/3 transfer from cytoplasmic to the nucleus, and inhibited the conversion of quiescent interstitial fibroblasts into myofibroblasts by blocking the nuclear translocation of Smad2/3 (Li et al., 2011). CPS-2, a polysaccharide extracted from *C. sinensis* could inhibit the PDGF-BB-induced proliferation of MCs through PDGF/ERK and TGF-β1/Smad signaling pathway, and inhibit the expression of α-SMA, PDGFRβ, TGF-β1, and Smad3, exerting the nephroprotection (Wang et al., 2014). Reversing EMT is a treatment option, which can slow the process of fibrosis and prevent the progression of DKD to ESRF, and the active components of *C. sinensis*, including cordycepin, have an advantage in mitigating renal fibrosis, especially early intervention.

### Regulation of Apoptosis

Apoptosis is one of the mechanisms of renal parenchymal cell reduction in the development of DKD, elimination of functional cells by apoptosis can induce renal insufficiency. In the progression of DKD, apoptosis can be induced by the increase of oxidative stress, and in the advanced DKD, with the progression of glomerular sclerosis, the number of apoptosis is increased and the renal function is gradually deteriorated (Sugiyama et al., 1996). It is indicated that *C. sinensis* can positively affect the homeostasis of apoptosis (Buenz et al., 2005), but the specific mechanism of regulating apoptosis in DKD remains to be explored. Klotho is a highly expressed anti-aging protein in kidney, which can inhibit oxidative stress by activating the Nrf2 signaling pathway, thereby protecting podocytes apoptosis in DKD (Xing et al., 2021). Capase-3 plays an important role in the process of apoptosis, and in the early stage of apoptosis, the activation of capase-3 can lyse the corresponding cytoplasmic nucleus substrate, eventually leading to apoptosis (Alnemri et al., 1996). TANG et al. demonstrated that the extracts of *C. sinensis* could suppress the apoptosis of NRK-52E cells by increasing the expression of Klotho, decreasing the expression of p53 and p21, and inhibiting the activation of capsase-3 (Tang et al., 2009). And *C. sinensis* had been shown could inhibit Hbx-induced apoptosis in HK-2 cells by suppressing the PI3K/Akt-Bcl-2 signaling cascades (He et al., 2020). Although it did not occur in DKD, the regulation of renal tubular epithelial cells apoptosis could still be used as a reference to lay the foundation for future research.

The pathological changes of DKD include mesangial proliferation, which is also an important reason for the increase of mesangial matrix and the abnormal synthesis and deposition of ECM (Lei et al., 2019). H1-A, a kind of polysaccharides extracted from *C. sinensis* was found to inhibit mesangial proliferation and protect MCs from cytolysis at high concentrations of DMSO, and possibly mediate the tyrosine phosphorylation of anti-apoptotic factors Bcl-2 and Bcl-XL to promote apoptosis (Yang et al., 2003). In contrast to inhibiting apoptosis in renal tubular epithelial cells, *C. sinensis* can promote apoptosis in MCs, which may be related to the pathology of DKD as thickening of the basement membrane and deposition of the mesangial matrix. Different cells have different pathological changes in the pathogenesis of DKD, and the effect of apoptosis has different meanings in different renal cells. In conclusion, it is necessary to continue to study the mechanism of *C. sinensis* regulating apoptosis in different renal intrinsic cells, and providing a new therapeutic target for the treatment of DKD.

## The Clinical Use of *Cordyceps sinensis* in Treating Diabetic Kidney Disease

At present, three major *cordyceps* preparations, Bailing capsule (BLC), Zhiling capsule (ZLC), and Jinshuibao capsule (JSBC) has been approved for clinical treatment of DKD in China (Luo et al., 2015).

Additional application of all three *cordyceps* preparations have been proved beneficial in treatment of DKD. Clinical studies have found that the combination of *cordyceps* preparations (BLC) with ACEI/ARB or SGLT2 inhibitors could significantly reduce the excretion of urinary protein, alleviate kidney damage, and delay the progression of DKD compared with the use of ACEI/ARB or SGLT2 inhibitors alone (Li Z. et al., 2019b; Li and Gao, 2021). In addition to improving kidney function, some scholars have made research on *cordyceps* preparations in improving inflammation, oxidative stress, and immune regulation of DKD patients. A previous study has shown that BLC was able to inhibit aldose reductase (AR), thereby inhibiting the polyol pathway in DKD (Luo et al., 2015). Specifically, Li et al. used BLC combined with dapagliflozin to treat patients with DKD for 12 weeks, the renal function indicators in the combination treatment group were significantly better than dapagliflozin alone, and the ocular vascular lesions of DKD patients were also alleviated (Li and Gao, 2021). Li et al. indicated that compared with the use of irbesartan alone, combined with BLC could significantly improve the micro-inflammatory state and oxidative stress response of DKD patients, with reduce levels of TNF-α, CRP, ROS and advanced protein oxidation product (AOPPS), and increased CD4^+^ and CD4^+^/CD8^+^ levels to regulate immune disorders (Li Z. et al., 2019b). JSBC combined with ACEI/ARB could effectively raise the total effective rate, reduce the 24 h urinary protein (24 h-UTP), urinary protein excretion rate and serum creatinine (Scr), and regulate glycolipid metabolism, the common using dose was from 3 to 6 capsules three times daily based on age (Li and Xu, 2020). The combination of JSBC and irbesartan could also effectively reduce endothelin-1 (ET-1), IL-6, TNF-α levels, and increase the expression of NO to improve vascular endothelial function and alleviate micro-inflammatory state in patients with DKD (Wu et al., 2019). Similarly, ZLC combined with telmisartan could significantly reduce the levels of Scr, 24 h-UTP, TNF-α, CRP, IL-6 and ET, increase NO levels, improve glomerular vasomotor function, and delay the progression of DKD (Wang and Qi, 2018). At present, relevant studies have not found adverse reactions to the prolonged use of *cordyceps* preparations (Sheng et al., 2020), but it still needs to be supported by more high-quality randomized controlled trials. And there is a lack of high-quality evidence on the occurrence of cardiovascular events associated with DKD (Li and Xu, 2020; Sheng et al., 2020).

Some researched aimed to compare the differences of three major *cordyceps* preparations. JSBC, BLC and ZLC are prepared by the fermentation of Paecilomyces hepiali (strain Cs-4), Hirsutella sinensis (strain Cs-C-Q80) and Mortierella SP, and 64, 39, and 56 components were identified in the essential oils of JSBC, BLC, and ZLC, respectively (Zhang et al., 2017). 5,6-Dihydro-6-pentyl-2H-pyran-2-one (massoia lactone) was the main component in the volatiles of JSBC, and pyrazinamide was characteristic in BLC. Besides, ZLC had the highest proportion of fatty acid compounds up to 27.1%, which could effectively enhance cell function and improved the metabolic capacity of cells (Zhang et al., 2017; Song et al., 2018). Meantime, the analysis of nucleosides and bases in fermented *cordyceps* preparations found that the content of guanosine, uridine and adenosine in ZLC was comparable, and it was higher than that in BLC and JSBC, while the 3 bases in BLC were the lowest (Zhang et al., 2009). However, in spite of the differences between compositions of 3 *cordyceps* preparations, no clinical studies are comparing the efficacy of the three preparations in the treatment of DKD, and whether there are different effects between them is still worth exploring. The differently using conditions of the 3 *cordyceps* preparations remain one of the problems that need to be explored in clinical and future experimental studies. It is worth exploring the conditions among the three preparations which are applied to DKD and whether there are differences between their effects, in order to facilitate the best application in clinic.

## Conclusion

**FIGURE 2:**
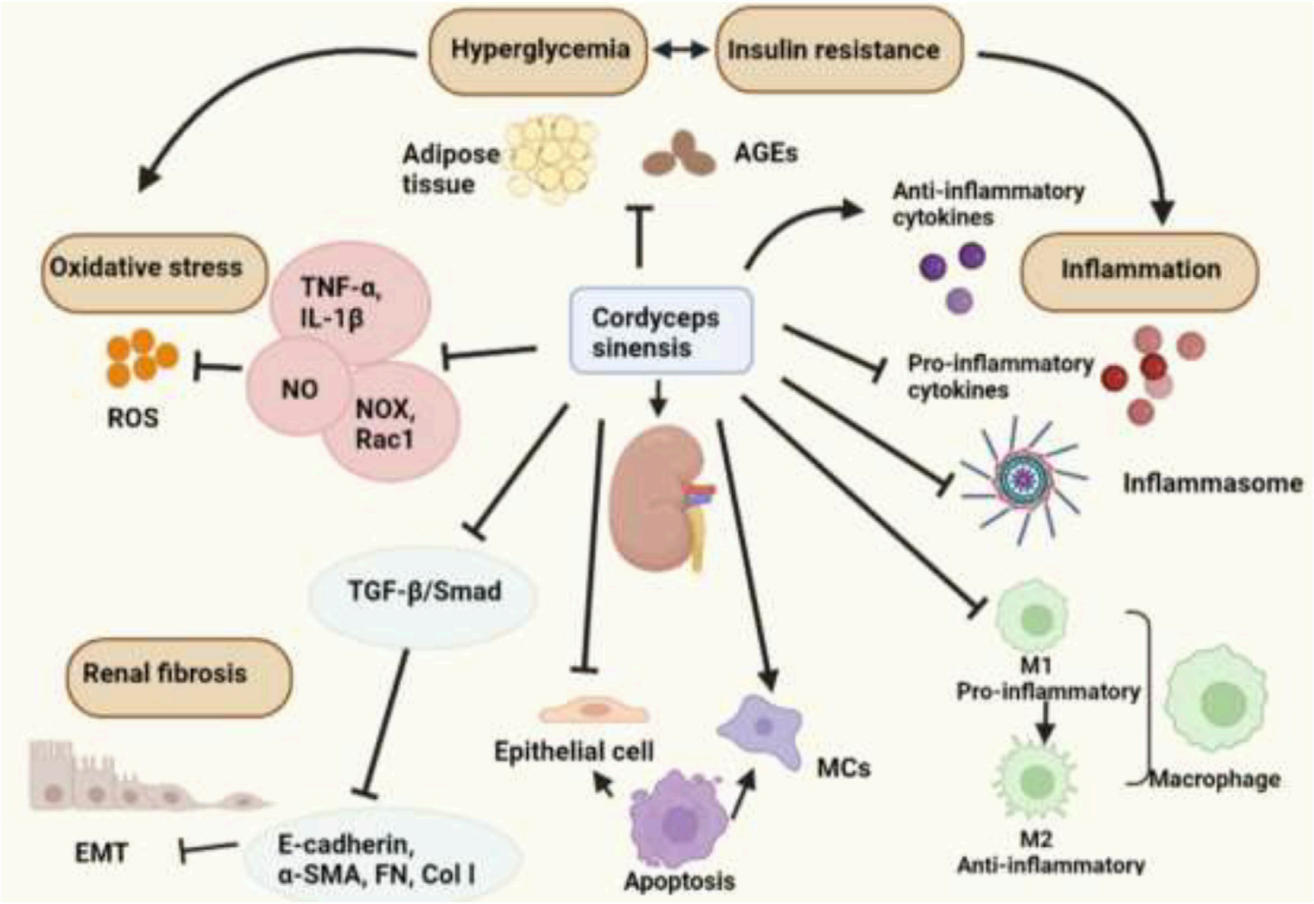
The biological activities of *Cordyceps sinensis* in treating DKD.

At present, the clinical treatment of CKD is focused on symptomatic treatment. The first-line treatments are mainly based on RAS blockers. However, there will be rising limitations in the use of drugs with the decline of renal function until it turns into ESRF. The emergence of Chinese herbal medicines and various Chinese patent medicines as the adjuvant drugs can effectively alleviate the side effects of drugs and slow the progression of kidney function. This review focused on mechanisms of *C. sinensis* and its extracts in the treatment of DKD (Table 2, Figure 2). Clinical applications and its efficacy of Chinese patent medicines made from *C. sinensis* in treatment of DKD were also reviewed.

**TABLE 2:** Major effects and targets of Cordyceps Sinensis in Diabetic Kidney Disease.

Effects	Materials	Subjects	Targets	Reference
Anti-hyperglycemia effects	CS-F10	STZ-induced and epinephrine-induced hyperglycemic mice	GLUT2	Kiho et al. (1999)
	β-sitosterol	high-fat diet and sucrose-induced type-2 diabetic rats	SOD, GST, CAT, GR, GSH, GP_X_, IRβ, GLUT4	Ponnulakshmi et al. (2019)
Anti-inflammatory effect	EPS	LPS-induced THP-1 cells, LPS-induced RAW264.7 cells, LPS-induced mice	NO, NF-κB, TNF-α, IL-1β, IL-10, iNOS	Li et al. (2020)
	CPS-F	PDGF-BB induced HMCs	ROS, TNF-α, TNF-R1, MCP-1, NOX1, PDGFRβ	Wang et al. (2015)
	*C. sinensis*	STZ-induced DKD rats, high-glucose exposed podocytes	P2X7R, NLRP3, ASC, caspase-1, IL-1β, IL-18, nephrin, podocin, WT-1, desmin	Wang et al. (2018)
	Cordycepin	Albumin-induced EMT in HK-2 cells	E-cadherin, α-SMA, ROS, NOX4, p22phox, p47phox, Rac1-GTP	Xiao et al. (2012)
Immunomodulatory effect	Intracellular polysaccharides	LPS-induced RAW264.7 cells	TNF-α, IL-6, IL-1β, IL-10, TGF-β1, iNOS, NO, ERK, JNK, p38, AKT	Liu et al. (2021)
	Cordycepin, adenosine	LPS-induced RAW264.7 cells	IL-1β, IL-6, TNF-α, RANTES, CX3CL1, IL-1ra, IL-10, TGF-β	Shin et al. (2009b)
	*C. sinensis*	Non-obese diabetic mice	CD4^+^CD25^+^FoxP3^+^ T reg cells, Th17 cells	Shi et al. (2009)
	Cordycepin	LPS-induced RAW264.7 cells	NO, IL-1β, IL-6, TNF-α, 11β-HSD1, PPARγ, ICAM-1, B7-1/-1	Shin et al. (2009a)
Anti-oxidant effect	CPS-F	PDGF-BB induced HMCs	ROS, TNF-α, TNFR1, MCP-1, NOX1, PDGFRβ	Wang et al. (2015)
	*C. sinensis* from India	LPS-induced THP1 cells	TNF-α, IL-1β, NO	Rathor et al. (2014)
	Cordycepin	Albumin-induced EMT in HK-2 cells	E-cadherin, α-SMA, ROS, NOX4, P22phox, P47phox, Rac1-GTP	Xiao et al. (2012)
Anti-fibrotic effect	nucleoside/nucleobase-rich extract	STZ-induced diabetic mice, high-glucose exposed HK-2 cells	E-cadherin, α-SMA, FN, Col Ⅰ, p38, ERK, JNK	Dong et al. (2019)
	*C. sinensis*	UUO rats	α-SMA, BAG3	Du et al. (2015)
	*C. sinensis*	5/6 subtotal nephrectomy rats	TGF-β1, Smad2, Smad3, TβRⅠ, TβRⅡ, α-SMA, FSP1, Smad7	Pan et al. (2013)
	Cordycepin	TGF-β1or BMP-4 induced NRK-52E cells/MCs, UUO mice	TGF-β, BMP-4, Col Ⅰ, ColⅣ, Smad1, Smad2, Smad3, eIF2α, CAGA, BRE	Gu et al. (2013)
	Cordycepin	TGF-β1 induced NRK-49F cells	α-SMA, FN, HGF, c-MET, TGF-β1, Smad2, Smad3	Li et al. (2011)
	CPS-2	PDGF-BB induced HMCs	α-SMA, PDGFRβ, TGF-β1, Smad3, ERK, TGFβR1	Wang et al. (2014)
Regulation of apoptosis	*C. sinensis*	Ang Ⅱ induced NRK-52E cells	Kl, p53, p21, caspase-3	Tang et al. (2009)
	*C. sinensis*	HBx induced HK-2 cells	Capse-3, capse-9, PI3K, Akt, Bax, Bcl-2	He et al. (2020)
	H1-A	IL-1 and PDGF-BB induced HMCs	Bcl-2, Bcl-XL	Yang et al. (2003)

However, some important problems should be further investigated. For example, in the regulation of apoptosis of *C. sinensis*, why different regulatory effects on different renal parenchymal cells remain to be explored. And the dose control of the extracted *C. sinensis* active substance should also be studied with a view to selecting the optimal clinical therapeutic dose. Moreover, the poly-component and multi-target effect of TCM is a double-edged sword. Although several main active ingredients, such as cordycepin, adenosine, and various polysaccharides, had been identified, there are still many components with unclear effects and functions. Therefore, it is suggested that we should continue to explore the potential active components and therapeutic effects of *C. sinensis* in the future. Pharmacological research on its main active extracts to candidate new agents and therapeutic targets are also imperative for its multiple applications in the clinic. Besides, considering the high prices of *C. sinensis*, more investigates are needed to reduce the economic burden on patients, including but not limited to artificial cultivation and development of new extraction methods. In addition, whether there are different effects among them still unclear and more research should be done to find the best conditions for each *cordyceps* preparation. High-quality prospective clinical trials with follow-up are also needed to provide convincing evidence of its safety and efficacy. We think that these explorations will provide new insight into clinical application of *C. sinensis* in treatment of DKD.

## References

[B135] Abou-Hany H. O. Atef H. Said E. Elkashef H. A. Salem H. A. (2018). Crocin Mediated Amelioration of Oxidative Burden and Inflammatory Cascade Suppresses Diabetic Nephropathy Progression in Diabetic Rats. Chemico-biol. interactions 284, 90–100. 10.1016/j.cbi.2018.02.001 29409856

[B1] Alicic R. Z. Rooney M. T. Tuttle K. R. (2017). Diabetic Kidney Disease: Challenges, Progress, and Possibilities. Clin. J. Am. Soc. Nephrol. 12, 2032–2045. 10.2215/CJN.11491116 28522654 PMC5718284

[B2] Alnemri E. S. Livingston D. J. Nicholson D. W. Salvesen G. Thornberry N. A. Wong W. W. (1996). Human ICE/CED-3 Protease Nomenclature. Cell. 87, 171. 10.1016/s0092-8674(00)81334-3 8861900

[B3] An J. Niu F. Sim J. J. (2021). Cardiovascular and Kidney Outcomes of Spironolactone or Eplerenone in Combination with ACEI/ARBs in Patients with Diabetic Kidney Disease. Pharmacotherapy 41, 998–1008. 10.1002/phar.2633 34655484

[B4] Artunc F. Schleicher E. Weigert C. Fritsche A. Stefan N. Häring H. U. (2016). The Impact of Insulin Resistance on the Kidney and Vasculature. Nat. Rev. Nephrol. 12, 721–737. 10.1038/nrneph.2016.145 27748389

[B5] Ashraf S. A. Elkhalifa A. E. O. Siddiqui A. J. Patel M. Awadelkareem A. M. Snoussi M. (2020). Cordycepin for Health and Wellbeing: A Potent Bioactive Metabolite of an Entomopathogenic Cordyceps Medicinal Fungus and its Nutraceutical and Therapeutic Potential. Molecules 25, 2735. 10.3390/molecules25122735 PMC7356751 32545666

[B6] Belska N. V. Guriev A. M. Danilets M. G. Trophimova E. S. Uchasova E. G. Ligatcheva A. A. (2010). Water-soluble Polysaccharide Obtained from Acorus calamus L. Classically Activates Macrophages and Stimulates Th1 Response. Int. Immunopharmacol. 10, 933–942. 10.1016/j.intimp.2010.05.005 20483383

[B7] Buenz E. J. Bauer B. A. Osmundson T. W. Motley T. J. (2005). The Traditional Chinese Medicine Cordyceps Sinensis and its Effects on Apoptotic Homeostasis. J. Ethnopharmacol. 96, 19–29. 10.1016/j.jep.2004.09.029 15588646

[B8] Cao C. Yang S. Zhou Z. (2020). The Potential Application of Cordyceps in Metabolic-Related Disorders. Phytother. Res. 34, 295–305. 10.1002/ptr.6536 31667949

[B9] Cao X. Zhang P. Yang J. (2007). Efficacy of Jinshuibao Combined with Valsartan in the Treatment of Early Type 2 Diabetic Nephropathy. Chin. J. New Drugs 16, 1303–1306.

[B10] Chang Y. Hsu W. H. Lu W. J. Jayakumar T. Liao J. C. Lin M. J. (2015). Inhibitory Mechanisms of CME-1, a Novel Polysaccharide from the Mycelia of Cordyceps Sinensis, in Platelet Activation. Curr. Pharm. Biotechnol. 16, 451–461. 10.2174/1389201016666150303152237 25751172

[B11] Chen G. Goeddel D. V. (2002). TNF-R1 Signaling: a Beautiful Pathway. Science 296, 1634–1635. 10.1126/science.1071924 12040173

[B12] Chen P. X. Wang S. Nie S. Marcone M. (2013). Properties of Cordyceps Sinensis: A Review. J. Funct. Foods 5, 550–569. 10.1016/j.jff.2013.01.034 32288794 PMC7104941

[B13] Chen W. Zhang W. Shen W. Wang K. (2010). Effects of the Acid Polysaccharide Fraction Isolated from a Cultivated Cordyceps Sinensis on Macrophages *In Vitro* . Cell. Immunol. 262, 69–74. 10.1016/j.cellimm.2010.01.001 20138259

[B14] Coward R. J. Welsh G. I. Yang J. Tasman C. Lennon R. Koziell A. (2005). The Human Glomerular Podocyte Is a Novel Target for Insulin Action. Diabetes 54, 3095–3102. 10.2337/diabetes.54.11.3095 16249431

[B15] Di Vincenzo A. Tana C. El Hadi H. Pagano C. Vettor R. Rossato M. (2019). Antioxidant, Anti-inflammatory, and Metabolic Properties of Tocopherols and Tocotrienols: Clinical Implications for Vitamin E Supplementation in Diabetic Kidney Disease. Int. J. Mol. Sci. 20, 5101. 10.3390/ijms20205101 PMC6834186 31618817

[B16] Dong C. Yao Y. (2012). Isolation, Characterization of Melanin Derived from Ophiocordyceps Sinensis, an Entomogenous Fungus Endemic to the Tibetan Plateau. J. Biosci. Bioeng. 113, 474–479. 10.1016/j.jbiosc.2011.12.001 22261188

[B17] Dong C. H. Yao Y. J. (2008). *In Vitro* evaluation of Antioxidant Activities of Aqueous Extracts from Natural and Cultured Mycelia of Cordyceps Sinensis. Leb. Wiss Technol. 41, 669–677. 10.1016/j.lwt.2007.05.002 PMC7126362 32287390

[B18] Dong Z. Sun Y. Wei G. Li S. Zhao Z. (2019). A Nucleoside/Nucleobase-Rich Extract from Cordyceps Sinensis Inhibits the Epithelial-Mesenchymal Transition and Protects against Renal Fibrosis in Diabetic Nephropathy. Molecules 24, 4119. 10.3390/molecules24224119 PMC6891521 31739543

[B19] Du F. Li S. Wang T. Zhang H. Y. Zong Z. H. Du Z. X. (2015). Cordyceps Sinensis Attenuates Renal Fibrosis and Suppresses BAG3 Induction in Obstructed Rat Kidney. Am. J. Transl. Res. 7, 932–940. 26175854 PMC4494144

[B20] Ekor M. (2014). The Growing Use of Herbal Medicines: Issues Relating to Adverse Reactions and Challenges in Monitoring Safety. Front. Pharmacol. 4, 177. 10.3389/fphar.2013.00177 24454289 PMC3887317

[B21] El Zahraa Z El Ashry F. Mahmoud M. F. El Maraghy N. N. Ahmed A. F. (2012). Effect of Cordyceps Sinensis and Taurine Either Alone or in Combination on Streptozotocin Induced Diabetes. Food Chem. Toxicol. 50, 1159–1165. 10.1016/j.fct.2011.12.020 22226943

[B22] Fitchett D. (2019). A Safety Update on Sodium Glucose Co-transporter 2 Inhibitors. Diabetes Obes. Metab. 21, 34–42. 10.1111/dom.13611 31081590

[B23] Glassock R. J. Warnock D. G. Delanaye P. (2017). The Global Burden of Chronic Kidney Disease: Estimates, Variability and Pitfalls. Nat. Rev. Nephrol. 13, 104–114. 10.1038/nrneph.2016.163 27941934

[B24] Gorin Y. Wauquier F. (2015). Upstream Regulators and Downstream Effectors of NADPH Oxidases as Novel Therapeutic Targets for Diabetic Kidney Disease. Mol. Cells 38, 285–296. 10.14348/molcells.2015.0010 25824546 PMC4400302

[B25] Gu L. Johno H. Nakajima S. Kato H. Takahashi S. Katoh R. (2013). Blockade of Smad Signaling by 3'-deoxyadenosine: a Mechanism for its Anti-fibrotic Potential. Lab. Investig. 93, 450–461. 10.1038/labinvest.2013.4 23439432

[B26] Haskó G. Szabó C. Németh Zh. Kvetan V. Pastores S. M. Vizi E. S. (1996). Adenosine Receptor Agonists Differentially Regulate IL-10, TNF-Alpha, and Nitric Oxide Production in RAW 264.7 Macrophages and in Endotoxemic Mice. J. Immunol. 157, 4634–4640. 8906843

[B27] He P. Lei J. Miao J. N. Wu D. Wang C. (2020). Cordyceps Sinensis Attenuates HBx-induced C-ell A-poptosis in HK-2 C-ells through S-uppressing the PI3K/Akt P-athway. Int. J. Mol. Med. 45, 1261–1269. 10.3892/ijmm.2020.4503 32124952

[B28] Hotamisligil G. S. Shargill N. S. Spiegelman B. M. (1993). Adipose Expression of Tumor Necrosis Factor-Alpha: Direct Role in Obesity-Linked Insulin Resistance. Science 259, 87–91. 10.1126/science.7678183 7678183

[B29] Hu H. Xiao L. Zheng B. Wei X. Ellis A. Liu Y. M. (2015). Identification of Chemical Markers in Cordyceps Sinensis by HPLC-MS/MS. Anal. Bioanal. Chem. 407, 8059–8066. 10.1007/s00216-015-8978-6 26302964 PMC4596796

[B30] Huang C. W. Hong T. W. Wang Y. J. Chen K. C. Pei J. C. Chuang T. Y. (2016). Ophiocordyceps Formosana Improves Hyperglycemia and Depression-like Behavior in an STZ-Induced Diabetic Mouse Model. BMC Complement. Altern. Med. 16, 310. 10.1186/s12906-016-1278-7 27553852 PMC4995616

[B31] Jha J. C. Banal C. Chow B. S. Cooper M. E. Jandeleit-Dahm K. (2016). Diabetes and Kidney Disease: Role of Oxidative Stress. Antioxid. Redox Signal 25, 657–684. 10.1089/ars.2016.6664 26906673 PMC5069735

[B32] Jia J. M. Ma X. C. Wu C. F. Wu L. J. Hu G. S. (2005). Cordycedipeptide A, a New Cyclodipeptide from the Culture Liquid of Cordyceps Sinensis (Berk.) Sacc. Chem. Pharm. Bull. (Tokyo) 53, 582–583. 10.1248/cpb.53.582 15863936

[B33] Jia J. M. Tao H. H. Feng B. M. (2009). Cordyceamides A and B from the Culture Liquid of Cordyceps Sinensis (BERK.) SACC. Chem. Pharm. Bull. (Tokyo) 57, 99–101. 10.1248/cpb.57.99 19122327

[B137] Jia G. Whaley-Connel A. Sowers J. R. (2018). Diabetic Cardiomyopathy: A Hyperglycaemia-And Insulin-Resistance-Induced Heart Disease. Diabetologia 61, 21–28. 10.1007/s00125-017-4390-4 28776083 PMC5720913

[B34] Kai Z. Yongjian L. Sheng G. Yu L. (2015). Effect of Dongchongxiacao (Cordyceps) Therapy on Contrast-Induced Nephropathy in Patients with Type 2 Diabetes and Renal Insufficiency Undergoing Coronary Angiography. J. Tradit. Chin. Med. 35, 422–427. 10.1016/s0254-6272(15)30119-9 26427112

[B35] Kanasaki K. Taduri G. Koya D. (2013). Diabetic Nephropathy: the Role of Inflammation in Fibroblast Activation and Kidney Fibrosis. Front. Endocrinol. (Lausanne) 4, 7. 10.3389/fendo.2013.00007 23390421 PMC3565176

[B36] Karalliedde J. Gnudi L. (2016). Diabetes Mellitus, a Complex and Heterogeneous Disease, and the Role of Insulin Resistance as a Determinant of Diabetic Kidney Disease. Nephrol. Dial. Transpl. 31, 206–213. 10.1093/ndt/gfu405 25550448

[B37] Kiho T. Hui J. Yamane A. Ukai S. (1993). Polysaccharides in Fungi. XXXII. Hypoglycemic Activity and Chemical Properties of a Polysaccharide from the Cultural Mycelium of Cordyceps Sinensis. Biol. Pharm. Bull. 16, 1291–1293. 10.1248/bpb.16.1291 8130781

[B38] Kiho T. Ookubo K. Usui S. Ukai S. Hirano K. (1999). Structural Features and Hypoglycemic Activity of a Polysaccharide (CS-F10) from the Cultured Mycelium of Cordyceps Sinensis. Biol. Pharm. Bull. 22, 966–970. 10.1248/bpb.22.966 10513622

[B39] Kim S. D. (2010). Isolation, Structure and Cholesterol Esterase Inhibitory Activity of a Polysaccharide, PS-A, from Cordyceps Sinensis. Jksabc 53, 784–789. 10.3839/jksabc.2010.118

[B40] Kim Y. S. Morgan M. J. Choksi S. Liu Z. G. (2007). TNF-induced Activation of the Nox1 NADPH Oxidase and its Role in the Induction of Necrotic Cell Death. Mol. Cell. 26, 675–687. 10.1016/j.molcel.2007.04.021 17560373

[B41] Kwon Y.-M. Cho S.-M. Kim J.-H. Lee J.-H. Lee Y.-A. Lee S.-J. (2001). Hypoglycemic Effect of Cordyceps Militaris. Korean J. Pharmacogn. 32, 327–329.

[B42] Lei D. Chengcheng L. Xuan Q. Yibing C. Lei W. Hao Y. (2019). Quercetin Inhibited Mesangial Cell Proliferation of Early Diabetic Nephropathy through the Hippo Pathway. Pharmacol. Res. 146, 104320. 10.1016/j.phrs.2019.104320 31220559

[B43] Li L. He D. Yang J. Wang X. (2011). Cordycepin Inhibits Renal Interstitial Myofibroblast Activation Probably by Inducing Hepatocyte Growth Factor Expression. J. Pharmacol. Sci. 117, 286–294. 10.1254/jphs.11127fp 22134049

[B44] Li L. Q. Song A. X. Yin J. Y. Siu K. C. Wong W. T. Wu J. Y. (2020). Anti-inflammation Activity of Exopolysaccharides Produced by a Medicinal Fungus Cordyceps Sinensis Cs-HK1 in Cell and Animal Models. Int. J. Biol. Macromol. 149, 1042–1050. 10.1016/j.ijbiomac.2020.02.022 32035153

[B45] Li S. P. Li P. Dong T. T. Tsim K. W. (2001). Anti-oxidation Activity of Different Types of Natural Cordyceps Sinensis and Cultured Cordyceps Mycelia. Phytomedicine 8, 207–212. 10.1078/0944-7113-00030 11417914

[B46] Li S. P. Zhang G. H. Zeng Q. Huang Z. G. Wang Y. T. Dong T. T. (2006). Hypoglycemic Activity of Polysaccharide, with Antioxidation, Isolated from Cultured Cordyceps Mycelia. Phytomedicine 13, 428–433. 10.1016/j.phymed.2005.02.002 16716913

[B47] Li X. Gao L. (2021). Effect of Bailing Capsule Combined with Dapagliflozin on Diabetic Nephropathy. Lishizhen Med. Materia Medica Res. 32, 355–357.

[B48] Li Y. Liu Y. Han X. Jin H. Ma S. (2019a). Arsenic Species in Cordyceps Sinensis and its Potential Health Risks. Front. Pharmacol. 10, 1471. 10.3389/fphar.2019.01471 31866869 PMC6910106

[B49] Li Y. Wang L. Xu B. Zhao L. Li L. Xu K. (2021). Based on Network Pharmacology Tools to Investigate the Molecular Mechanism of Cordyceps Sinensis on the Treatment of Diabetic Nephropathy. J. Diabetes Res. 2021, 8891093. 10.1155/2021/8891093 33628839 PMC7884116

[B50] Li Y. Xu G. (2020). Clinical Efficacy and Safety of Jinshuibao Combined with ACEI/ARB in the Treatment of Diabetic Kidney Disease: A Meta-Analysis of Randomized Controlled Trials. J. Ren. Nutr. 30, 92–100. 10.1053/j.jrn.2019.03.083 31201013

[B51] Li Z. Zhang G. Zheng S. Zheng J. Zhang Y. Yuan P. (2019b). Effects of Bailing Capsule Combined with Irbesartan on Oxidative Stress, Inflammatory Response and Immune Function in Patients with Diabetic Nephropathy. J. Hainan Med. Univ. 25, 670–673. 10.13210/j.cnki.jhmu.20190322.004

[B52] Liu J. Li Y. Zan K. Zheng J. Guo L. Ma S. (2016). A Comparative Study on the Content of Pb, Cd, as, Hg and Cu between Cultivated and Natural Cordyceps Sinensis. Chin. Pharm. Affa 30, 912–918. 10.16153/j.1002-7777.2016.09.012

[B53] Liu Y. Li Q. Z. Li L. D. Zhou X. W. (2021). Immunostimulatory Effects of the Intracellular Polysaccharides Isolated from Liquid Culture of Ophiocordyceps Sinensis (Ascomycetes) on RAW264.7 Cells via the MAPK and PI3K/Akt Signaling Pathways. J. Ethnopharmacol. 275, 114130. 10.1016/j.jep.2021.114130 33892066

[B54] Liu Y. Wang J. Wang W. Zhang H. Zhang X. Han C. (2015). The Chemical Constituents and Pharmacological Actions of Cordyceps Sinensis. Evid. Based Complement. Altern. Med. 2015, 575063. 10.1155/2015/575063 PMC4415478 25960753

[B55] Lo H. C. Hsu T. H. Tu S. T. Lin K. C. (2006). Anti-hyperglycemic Activity of Natural and Fermented Cordyceps Sinensis in Rats with Diabetes Induced by Nicotinamide and Streptozotocin. Am. J. Chin. Med. 34, 819–832. 10.1142/S0192415X06004314 17080547

[B56] Lo H. C. Tu S. T. Lin K. C. Lin S. C. (2004). The Anti-hyperglycemic Activity of the Fruiting Body of Cordyceps in Diabetic Rats Induced by Nicotinamide and Streptozotocin. Life Sci. 74, 2897–2908. 10.1016/j.lfs.2003.11.003 15050427

[B57] Lu Q. Li C. Chen W. Shi Z. Zhan R. He R. (2018). Clinical Efficacy of Jinshuibao Capsules Combined with Angiotensin Receptor Blockers in Patients with Early Diabetic Nephropathy: A Meta-Analysis of Randomized Controlled Trials. Evid. Based Complement. Altern. Med. 2018, 6806943. 10.1155/2018/6806943 PMC5941802 29849721

[B58] Lu Z. Zhong Y. Liu W. Xiang L. Deng Y. (2019). The Efficacy and Mechanism of Chinese Herbal Medicine on Diabetic Kidney Disease. J. Diabetes Res. 2019, 2697672. 10.1155/2019/2697672 31534972 PMC6732610

[B59] Luo Y. Yang S. K. Zhou X. Wang M. Tang D. Liu F. Y. (2015). Use of Ophiocordyceps Sinensis (Syn. Cordyceps Sinensis) Combined with Angiotensin-Converting Enzyme Inhibitors (ACEI)/angiotensin Receptor Blockers (ARB) versus ACEI/ARB Alone in the Treatment of Diabetic Kidney Disease: a Meta-Analysis. Ren. Fail 37, 614–634. 10.3109/0886022X.2015.1009820 25682973

[B60] Mantovani A. Sica A. Locati M. (2007). New Vistas on Macrophage Differentiation and Activation. Eur. J. Immunol. 37, 14–16. 10.1002/eji.200636910 17183610

[B61] Matsuda H. Akaki J. Nakamura S. Okazaki Y. Kojima H. Tamesada M. (2009). Apoptosis-inducing Effects of Sterols from the Dried Powder of Cultured Mycelium of Cordyceps Sinensis. Chem. Pharm. Bull. (Tokyo) 57, 411–414. 10.1248/cpb.57.411 19336939

[B62] Mayer G. J. Wanner C. Weir M. R. Inzucchi S. E. Koitka-Weber A. Hantel S. (2019). Analysis from the EMPA-REG OUTCOME® Trial indicates Empagliflozin May Assist in Preventing the progression of Chronic Kidney Disease in Patients with Type 2 Diabetes Irrespective of Medications that Alter Intrarenal Hemodynamics. Kidney Int. 96, 489–504. 10.1016/j.kint.2019.02.033 31142441

[B63] Menzies R. I. Booth J. W. R. Mullins J. J. Bailey M. A. Tam F. W. K. Norman J. T. (2017). Hyperglycemia-induced Renal P2X7 Receptor Activation Enhances Diabetes-Related Injury. EBioMedicine 19, 73–83. 10.1016/j.ebiom.2017.04.011 28434946 PMC5440600

[B64] Műzes G. Molnár B. Tulassay Z. Sipos F. (2012). Changes of the Cytokine Profile in Inflammatory Bowel Diseases. World J. Gastroenterol. 18, 5848–5861. 10.3748/wjg.v18.i41.5848 23139600 PMC3491591

[B65] Niu Y. J. Tao R. Y. Liu Q. Tian J. Y. Ye F. Zhu P. (2010). Improvement on Lipid Metabolic Disorder by 3'-deoxyadenosine in High-Fat-Diet-Induced Fatty Mice. Am. J. Chin. Med. 38, 1065–1075. 10.1142/S0192415X10008470 21061461

[B66] Olatunji O. J. Tang J. Tola A. Auberon F. Oluwaniyi O. Ouyang Z. (2018). The Genus Cordyceps: An Extensive Review of its Traditional Uses, Phytochemistry and Pharmacology. Fitoterapia 129, 293–316. 10.1016/j.fitote.2018.05.010 29775778

[B67] Olefsky J. M. Glass C. K. (2010). Macrophages, Inflammation, and Insulin Resistance. Annu. Rev. Physiol. 72, 219–246. 10.1146/annurev-physiol-021909-135846 20148674

[B68] Oyarzún C. Garrido W. Alarcón S. Yáñez A. Sobrevia L. Quezada C. (2017). Adenosine Contribution to Normal Renal Physiology and Chronic Kidney Disease. Mol. Asp. Med. 55, 75–89. 10.1016/j.mam.2017.01.004 28109856

[B69] Pan M. M. Zhang M. H. Ni H. F. Chen J. F. Xu M. Phillips A. O. (2013). Inhibition of TGF-β1/Smad Signal Pathway Is Involved in the Effect of Cordyceps Sinensis against Renal Fibrosis in 5/6 Nephrectomy Rats. Food Chem. Toxicol. 58, 487–494. 10.1016/j.fct.2013.04.037 23624380

[B70] Parcell A. C. Smith J. M. Schulthies S. S. Myrer J. W. Fellingham G. (2004). Cordyceps Sinensis (CordyMax Cs-4) Supplementation Does Not Improve Endurance Exercise Performance. Int. J. Sport Nutr. Exerc Metab. 14, 236–242. 10.1123/ijsnem.14.2.236 15118196

[B71] Paterson R. R. (2008). Cordyceps: a Traditional Chinese Medicine and Another Fungal Therapeutic Biofactory? Phytochemistry 69, 1469–1495. 10.1016/j.phytochem.2008.01.027 18343466 PMC7111646

[B72] Peleli M. Carlstrom M. (2017). Adenosine Signaling in Diabetes Mellitus and Associated Cardiovascular and Renal Complications. Mol. Asp. Med. 55, 62–74. 10.1016/j.mam.2016.12.001 28089906

[B73] Pichler R. Afkarian M. Dieter B. P. Tuttle K. R. (2017). Immunity and Inflammation in Diabetic Kidney Disease: Translating Mechanisms to Biomarkers and Treatment Targets. Am. J. Physiol. Ren. Physiol. 312, F716–f731. 10.1152/ajprenal.00314.2016 PMC6109808 27558558

[B74] Ponnulakshmi R. Shyamaladevi B. Vijayalakshmi P. Selvaraj J. (2019). In Silico and *In Vivo* Analysis to Identify the Antidiabetic Activity of Beta Sitosterol in Adipose Tissue of High Fat Diet and Sucrose Induced Type-2 Diabetic Experimental Rats. Toxicol. Mech. Methods 29, 276–290. 10.1080/15376516.2018.1545815 30461321

[B75] Qian G. M. Pan G. F. Guo J. Y. (2012). Anti-inflammatory and Antinociceptive Effects of Cordymin, a Peptide Purified from the Medicinal Mushroom Cordyceps Sinensis. Nat. Prod. Res. 26, 2358–2362. 10.1080/14786419.2012.658800 22348255

[B76] Rathor R. Mishra K. P. Pal M. Amitabh S. Vats P. Kirar V. (2014). Scientific Validation of the Chinese Caterpillar Medicinal Mushroom, Ophiocordyceps Sinensis (Ascomycetes) from India: Immunomodulatory and Antioxidant Activity. Int. J. Med. Mushrooms 16, 541–553. 10.1615/intjmedmushrooms.v16.i6.40 25404219

[B77] Reidy K. Kang H. M. Hostetter T. Susztak K. (2014). Molecular Mechanisms of Diabetic Kidney Disease. J. Clin. Investig. 124, 2333–2340. 10.1172/JCI72271 24892707 PMC4089448

[B78] Rotter V. Nagaev I. Smith U. (2003). Interleukin-6 (IL-6) Induces Insulin Resistance in 3T3-L1 Adipocytes and Is, like IL-8 and Tumor Necrosis Factor-Alpha, Overexpressed in Human Fat Cells from Insulin-Resistant Subjects. J. Biol. Chem. 278, 45777–45784. 10.1074/jbc.M301977200 12952969

[B79] Sabapathy V. Stremska M. E. Mohammad S. Corey R. L. Sharma P. R. Sharma R. (2019). Novel Immunomodulatory Cytokine Regulates Inflammation, Diabetes, and Obesity to Protect from Diabetic Nephropathy. Front. Pharmacol. 10, 572. 10.3389/fphar.2019.00572 31191312 PMC6540785

[B80] Sailaja Devi M. M. Suresh Y. Das U. N. (2000). M., Suresh, Y. & DasPreservation of the Antioxidant Status in Chemically-Induced Diabetes Mellitus by Melatonin. J. Pineal Res. 29, 108–115. 10.1034/j.1600-079x.2000.290207.x 10981824

[B81] Sarafidis P. Ferro C. J. Morales E. Ortiz A. Malyszko J. Hojs R. (2019). SGLT-2 Inhibitors and GLP-1 Receptor Agonists for Nephroprotection and Cardioprotection in Patients with Diabetes Mellitus and Chronic Kidney Disease. A Consensus Statement by the EURECA-M and the DIABESITY Working Groups of the ERA-EDTA. Nephrol. Dial. Transpl. 34, 208–230. 10.1093/ndt/gfy407 30753708

[B82] Sedeek M. Nasrallah R. Touyz R. M. Hébert R. L. (2013). NADPH Oxidases, Reactive Oxygen Species, and the Kidney: Friend and Foe. J. Am. Soc. Nephrol. 24, 1512–1518. 10.1681/ASN.2012111112 23970124 PMC3785272

[B83] Shahzad K. Bock F. Dong W. Wang H. Kopf S. Kohli S. (2015). Nlrp3-inflammasome Activation in Non-myeloid-derived Cells Aggravates Diabetic Nephropathy. Kidney Int. 87, 74–84. 10.1038/ki.2014.271 25075770 PMC4284813

[B84] Shashidhar M. G. Giridhar P. Udaya Sankar K. Manohar B. (2013). Bioactive Principles from Cordyceps Sinensis: A Potent Food Supplement - A Review. J. Funct. Foods 5, 1013–1030. 10.1016/j.jff.2013.04.018 32288795 PMC7104994

[B85] Sheng X. Dong Y. Cheng D. Wang N. Guo Y. (2020). Efficacy and Safety of Bailing Capsules in the Treatment of Type 2 Diabetic Nephropathy: a Meta-Analysis. Ann. Palliat. Med. 9, 3885–3898. 10.21037/apm-20-1799 33222468

[B86] Shi B. Wang Z. Jin H. Chen Y. W. Wang Q. Qian Y. (2009). Immunoregulatory Cordyceps Sinensis Increases Regulatory T Cells to Th17 Cell Ratio and Delays Diabetes in NOD Mice. Int. Immunopharmacol. 9, 582–586. 10.1016/j.intimp.2009.01.030 19557879

[B87] Shin S. Lee S. Kwon J. Moon S. Lee S. Lee C. K. (2009a). Cordycepin Suppresses Expression of Diabetes Regulating Genes by Inhibition of Lipopolysaccharide-Induced Inflammation in Macrophages. Immune Netw. 9, 98–105. 10.4110/in.2009.9.3.98 20107539 PMC2803303

[B88] Shin S. Moon S. Park Y. Kwon J. Lee S. Lee C. K. (2009b). Role of Cordycepin and Adenosine on the Phenotypic Switch of Macrophages via Induced Anti-inflammatory Cytokines. Immune Netw. 9, 255–264. 10.4110/in.2009.9.6.255 20157613 PMC2816959

[B89] Sidhom E. H. Kim C. Kost-Alimova M. Ting M. T. Keller K. Avila-Pacheco J. (2021). Targeting a Braf/Mapk Pathway Rescues Podocyte Lipid Peroxidation in CoQ-Deficiency Kidney Disease. J. Clin. Investig. 131, e141380. 10.1172/JCI141380 PMC7919729 33444290

[B90] Snel M. Jonker J. T. Schoones J. Lamb H. De Roos A. Pijl H. (2012). Ectopic Fat and Insulin Resistance: Pathophysiology and Effect of Diet and Lifestyle Interventions. Int. J. Endocrinol. 2012, 983814. 10.1155/2012/983814 22675355 PMC3366269

[B91] Soldatos G. Cooper M. E. (2008). Diabetic Nephropathy: Important Pathophysiologic Mechanisms. Diabetes Res. Clin. Pract. 82, S75–S79. 10.1016/j.diabres.2008.09.042 18994672

[B92] Song Y. Hu P. Zhao S. Zhang H. Jiang Z. Wang Y. (2018). Distinction of Fermented Cordyceps Products from Different Strains and Analysis of Their Volatile Components by HS-SPME/GC-MS Combined Wtih Chemometrics Analysis. Chin. J. Pharm. Analysis 38, 67–78. 10.16155/j.0254-1793.2018.01.09

[B93] Srivastava S. P. Hedayat A. F. Kanasaki K. Goodwin J. E. (2019). microRNA Crosstalk Influences Epithelial-To-Mesenchymal, Endothelial-To-Mesenchymal, and Macrophage-To-Mesenchymal Transitions in the Kidney. Front. Pharmacol. 10, 904. 10.3389/fphar.2019.00904 31474862 PMC6707424

[B94] Su L. J. Zhang J. H. Gomez H. Murugan R. Hong X. Xu D. (2019). Reactive Oxygen Species-Induced Lipid Peroxidation in Apoptosis, Autophagy, and Ferroptosis. Oxid. Med. Cell. Longev. 2019, 5080843. 10.1155/2019/5080843 31737171 PMC6815535

[B95] Sugiyama H. Kashihara N. Makino H. Yamasaki Y. Ota A. (1996). Apoptosis in Glomerular Sclerosis. Kidney Int. 49, 103–111. 10.1038/ki.1996.14 8770955

[B96] Tang R. Zhou Q. Shu J. Tang T. Ao X. Peng W. (2009). Effect of Cordyceps Sinensis Extract on Klotho Expression and Apoptosis in Renal Tubular Epithelial Cells Induced by Angiotensin II. Zhong Nan Da Xue Xue Bao Yi Xue Ban. 34, 300–307. 19411745

[B97] Tang S. C. W. Yiu W. H. (2020). Innate Immunity in Diabetic Kidney Disease. Nat. Rev. Nephrol. 16, 206–222. 10.1038/s41581-019-0234-4 31942046

[B98] Tilg H. Moschen A. R. (2006). Adipocytokines: Mediators Linking Adipose Tissue, Inflammation and Immunity. Nat. Rev. Immunol. 6, 772–783. 10.1038/nri1937 16998510

[B136] Tomás E. Lin Y. S. Dagher Z. Saha A. Luo Z. Ido Y. (2002). Hyperglycemia and Insulin Resistance: Possible Mechanisms. Ann. N Y. Acad. Sci. 967, 43–51. 10.1111/j.1749-6632.2002.tb04262.x 12079834

[B99] Tuli H. S. Sandhu S. S. Sharma A. K. (2014). Pharmacological and Therapeutic Potential of Cordyceps with Special Reference to Cordycepin. 3 Biotech. 4, 1–12. 10.1007/s13205-013-0121-9 PMC3909570 28324458

[B100] Tuttle K. R. Bakris G. L. Bilous R. W. Chiang J. L. de Boer I. H. (2014). Diabetic Kidney Disease: a Report from an ADA Consensus Conference. Diabetes Care 37, 2864–2883. 10.2337/dc14-1296 25249672 PMC4170131

[B101] Urner S. Ho F. Jha J. C. Ziegler D. Jandeleit-Dahm K. (2020). NADPH Oxidase Inhibition: Preclinical and Clinical Studies in Diabetic Complications. Antioxid. Redox Signal 33, 415–434. 10.1089/ars.2020.8047 32008354

[B102] Vonend O. Turner C. M. Chan C. M. Loesch A. Dell'anna G. C. Srai K. S. (2004). Glomerular Expression of the ATP-Sensitive P2X Receptor in Diabetic and Hypertensive Rat Models. Kidney Int. 66, 157–166. 10.1111/j.1523-1755.2004.00717.x 15200422

[B103] Wada J. Makino H. (2013). Inflammation and the Pathogenesis of Diabetic Nephropathy. Clin. Sci. (Lond) 124, 139–152. 10.1042/CS20120198 23075333

[B104] Wang C. Hou X. X. Rui H. L. Li L. J. Zhao J. Yang M. (2018). Artificially Cultivated Ophiocordyceps Sinensis Alleviates Diabetic Nephropathy and its Podocyte Injury via Inhibiting P2X7R Expression and NLRP3 Inflammasome Activation. J. Diabetes Res. 2018, 1390418. 10.1155/2018/1390418 30534570 PMC6252193

[B105] Wang M. Kornsakulkarn J. Srichomthong K. Feng T. Liu J. K. Isaka M. (2019). Antimicrobial Anthraquinones from Cultures of the Ant Pathogenic Fungus Cordyceps Morakotii BCC 56811. J. Antibiot. (Tokyo) 72, 141–147. 10.1038/s41429-018-0135-y 30622295

[B106] Wang W. Zhang X. N. Yin H. Li X. B. Hu X. P. Liu H. (2013). Effects of Bailing Capsules for Renal Transplant Recipients: a Retrospective Clinical Study. Chin. Med. J. Engl. 126, 1895–1899. 23673106

[B107] Wang W. Qi J. (2018). Clinical Study of Zhiling Capsule Combined with Telmisartan in Treatment of Early Diabetic Nephropathy. Drugs & Clin. 33, 1494–1497.

[B108] Wang Y. Liu D. Zhao H. Jiang H. Luo C. Wang M. (2014). Cordyceps Sinensis Polysaccharide CPS-2 Protects Human Mesangial Cells from PDGF-BB-Induced Proliferation through the PDGF/ERK and TGF-β1/Smad Pathways. Mol. Cell. Endocrinol. 382, 979–988. 10.1016/j.mce.2013.11.018 24309234

[B109] Wang Y. Wang Y. Liu D. Wang W. Zhao H. Wang M. (2015). Cordyceps Sinensis Polysaccharide Inhibits PDGF-BB-Induced Inflammation and ROS Production in Human Mesangial Cells. Carbohydr. Polym. 125, 135–145. 10.1016/j.carbpol.2015.02.012 25857968

[B139] Wei Y. Li M. Yu J. (2019). Research Progress in the Relationship Between Chronic Inflammation and Insulin Resistance. J. Clinical Pathol. Res. 39, 640–645. 10.3978/j.issn.2095-6959.2019.03.030

[B110] Whaley-Connell A. T. Morris E. M. Rehmer N. Yaghoubian J. C. Wei Y. Hayden M. R. (2007). Albumin Activation of NAD(P)H Oxidase Activity Is Mediated via Rac1 in Proximal Tubule Cells. Am. J. Nephrol. 27, 15–23. 10.1159/000098432 17204833

[B111] Wheeler D. C. Stefánsson B. V. Jongs N. Chertow G. M. Greene T. Hou F. F. (2021). Effects of Dapagliflozin on Major Adverse Kidney and Cardiovascular Events in Patients with Diabetic and Non-diabetic Chronic Kidney Disease: a Prespecified Analysis from the DAPA-CKD Trial. Lancet Diabetes & Endocrinol. 9, 22–31. 10.1016/s2213-8587(20)30369-7 33338413

[B112] Wotton D. Massagué J. (2001). Smad Transcriptional Corepressors in TGF Beta Family Signaling. Curr. Top. Microbiol. Immunol. 254, 145–164. 10.1007/978-3-662-10595-5_8 11190572

[B113] Wu H. Wang Y. Hu F. Cao J. (2019). Clinical Effects of Jinshuibao Capsules Combined with Irbesartan on Patients with Diabetic Nephropathy. Chin. Tradit. Pat. Med. 41, 75–78.

[B114] Wu Y. Hu N. Pan Y. Zhou L. Zhou X. (2007). Isolation and Characterization of a Mannoglucan from Edible Cordyceps Sinensis Mycelium. Carbohydr. Res. 342, 870–875. 10.1016/j.carres.2007.01.005 17258695

[B115] Xiao L. Ge Y. Sun L. Xu X. Xie P. Zhan M. (2012). Cordycepin Inhibits Albumin-Induced Epithelial-Mesenchymal Transition of Renal Tubular Epithelial Cells by Reducing Reactive Oxygen Species Production. Free Radic. Res. 46, 174–183. 10.3109/10715762.2011.647688 22149621

[B116] Xing L. Guo H. Meng S. Zhu B. Fang J. Huang J. (2021). Klotho Ameliorates Diabetic Nephropathy by Activating Nrf2 Signaling Pathway in Podocytes. Biochem. Biophys. Res. Commun. 534, 450–456. 10.1016/j.bbrc.2020.11.061 33256980

[B117] Xu B. H. Sheng J. You Y. K. Huang X. R. Ma R. C. W. Wang Q. (2020). Deletion of Smad3 Prevents Renal Fibrosis and Inflammation in Type 2 Diabetic Nephropathy. Metabolism 103, 154013. 10.1016/j.metabol.2019.154013 31734275

[B118] Yalin W. Ishurd O. Cuirong S. Yuanjiang P. (2005). Structure Analysis and Antitumor Activity of (1-->3)BbetaDdGglucans (Cordyglucans) from the Mycelia of Cordyceps Sinensis. Planta Med. 71, 381–384. 10.1055/s-2005-864111 15856422

[B119] Yan J. K. Wang W. Q. Wu J. Y. (2014). Recent Advances in Cordyceps Sinensis Polysaccharides: Mycelial Fermentation, Isolation, Structure, and Bioactivities: A Review. J. Funct. Foods 6, 33–47. 10.1016/j.jff.2013.11.024 32362940 PMC7185505

[B138] Yan T. Nian T. Li F. He B. Jia Y. Bi K. (2020). Salidroside From Rhodiola Wallichiana var. Cholaensis Reverses Insulin Resistance and Stimulates the GLP-1 Secretion by Alleviating ROS-Mediated Activation of MAPKs Signaling Pathway and Mitigating Apoptosis. J. Food Biochem. 44, e13446. 10.1111/jfbc.13446 32910486

[B120] Yang L. Y. Huang W. J. Hsieh H. G. Lin C. Y. (2003). H1-A Extracted from Cordyceps Sinensis Suppresses the Proliferation of Human Mesangial Cells and Promotes Apoptosis, Probably by Inhibiting the Tyrosine Phosphorylation of Bcl-2 and Bcl-XL. J. Lab. Clin. Med. 141, 74–83. 10.1067/mlc.2003.6 12518171

[B121] Yang P. Zhao X. X. Zhang Y. W. (2018). Comparison and Review on Specifications of Fermented Cordyceps Sinensis Products. Zhongguo Zhong Yao Za Zhi 43, 463–468. 10.19540/j.cnki.cjcmm.20171012.001 29600609

[B122] Yin M. Zhang Y. Li H. (2019). Advances in Research on Immunoregulation of Macrophages by Plant Polysaccharides. Front. Immunol. 10, 145. 10.3389/fimmu.2019.00145 30804942 PMC6370632

[B123] Yoon S. Y. Park S. J. Park Y. J. (2018). The Anticancer Properties of Cordycepin and Their Underlying Mechanisms. Int. J. Mol. Sci. 19, 3027. 10.3390/ijms19103027 PMC6212910 30287757

[B124] Yu H. M. Wang B. S. Huang S. C. Duh P. D. (2006). Comparison of Protective Effects between Cultured Cordyceps Militaris and Natural Cordyceps Sinensis against Oxidative Damage. J. Agric. Food Chem. 54, 3132–3138. 10.1021/jf053111w 16608242

[B125] Yue K. Ye M. Zhou Z. Sun W. Lin X. (2013). The Genus Cordyceps: a Chemical and Pharmacological Review. J. Pharm. Pharmacol. 65, 474–493. 10.1111/j.2042-7158.2012.01601.x 23488776

[B126] Zatz R. Dunn B. R. Meyer T. W. Anderson S. Rennke H. G. Brenner B. M. (1986). Prevention of Diabetic Glomerulopathy by Pharmacological Amelioration of Glomerular Capillary Hypertension. J. Clin. Investig. 77, 1925–1930. 10.1172/JCI112521 3011862 PMC370553

[B127] Zhang G. Huang Y. Bian Y. Wong J. H. Ng T. B. Wang H. (2006). Hypoglycemic Activity of the Fungi Cordyceps Militaris, Cordyceps Sinensis, Tricholoma Mongolicum, and Omphalia Lapidescens in Streptozotocin-Induced Diabetic Rats. Appl. Microbiol. Biotechnol. 72, 1152–1156. 10.1007/s00253-006-0411-9 16575562

[B128] Zhang H. Li Y. Mi J. Zhang M. Wang Y. Jiang Z. (2017). GC-MS Profiling of Volatile Components in Different Fermentation Products of Cordyceps Sinensis Mycelia. Molecules 22, 1800. 10.3390/molecules22101800 PMC6151420 29064460

[B129] Zhang P. Xiao X. Li Y. Lin R. (2009). Chemical Analysis of Nucleosides and Alkalines Components in Five Preparations of Fermental Cordyceps. Chin. J. Pharm. Analysis, 889.

[B130] Zhang W. Yang J. Chen J. Hou Y. Han X. (2005a). Immunomodulatory and Antitumour Effects of an Exopolysaccharide Fraction from Cultivated Cordyceps Sinensis (Chinese Caterpillar Fungus) on Tumour-Bearing Mice. Biotechnol. Appl. Biochem. 42, 9–15. 10.1042/BA20040183 15574120

[B131] Zhang Y. Handy D. E. Loscalzo J. (2005b). Adenosine-dependent Induction of Glutathione Peroxidase 1 in Human Primary Endothelial Cells and Protection against Oxidative Stress. Circ. Res. 96, 831–837. 10.1161/01.RES.0000164401.21929.CF 15802613

[B132] Zhou L. Wang S. Hao Q. Kang L. Kang C. Yang J. (2018). Bioaccessibility and Risk Assessment of Heavy Metals, and Analysis of Arsenic Speciation in Cordyceps Sinensis. Chin. Med. 13, 40. 10.1186/s13020-018-0196-7 30083223 PMC6069848

[B133] Zhu J. S. Halpern G. M. Jones K. (1998). The Scientific Rediscovery of a Precious Ancient Chinese Herbal Regimen: Cordyceps Sinensis: Part II. J. Altern. Complement. Med. 4, 429–457. 10.1089/acm.1998.4.429 9884180

[B134] Zhu L. Fu X. Chen X. Han X. Dong P. (2017). M2 Macrophages Induce EMT through the TGF-β/Smad2 Signaling Pathway. Cell. Biol. Int. 41, 960–968. 10.1002/cbin.10788 28493530

